# Racial disparities in extended venous thromboembolism prophylaxis after hysterectomy

**DOI:** 10.1371/journal.pone.0318433

**Published:** 2025-01-28

**Authors:** Wenbo Wu, Sherry Wu, Sim Berlene Mariano, Richard E. Burney, Jonathan P. Kuriakose

**Affiliations:** 1 Departments of Population Health and Medicine, New York University Grossman School of Medicine, New York, NY, United States of America; 2 Center for Data Science, New York University, New York, NY, United States of America; 3 Department of Biostatistics, New York University School of Global Public Health, New York, NY, United States of America; 4 Quantitative Biomedical Sciences Program, Geisel School of Medicine, Dartmouth College, Hanover, NH, United States of America; 5 Robert Wood Johnson Medical School, Rutgers University, New Brunswick, NJ, United States of America; 6 Department of Obstetrics & Gynecology, New York University Long Island School of Medicine, Mineola, NY, United States of America; 7 Michigan Surgical Quality Collaborative, Institute of Health Policy & Innovation, Ann Arbor, MI, United States of America; 8 Department of Surgery, Michigan Medicine, Ann Arbor, MI, United States of America; 9 Department of Otolaryngology–Head & Neck Surgery, Northwestern University Feinberg School of Medicine, Chicago, IL, United States of America; Albert Einstein College of Medicine / NYCHH-Jacobi Medical Center, UNITED STATES OF AMERICA

## Abstract

**Background:**

Venous thromboembolism (VTE) is a significant preventable cause of postoperative morbidity and mortality after major abdominopelvic surgery that calls for extended VTE prophylaxis (eVTEp). Literature suggests that significant racial disparities may exist in post-operative care.

**Objective:**

The study sought to examine if racial disparities exist in the administration of eVTEp after hysterectomy in a statewide collaborative.

**Methods:**

We conducted a retrospective cohort study of post-hysterectomy patients across 69 hospitals in the Michigan Surgical Quality Collaborative from January 2016 to February 2020. The variable of interest was race (Black/African or White American). The primary outcome was administration or absence of eVTEp. Descriptive statistics and mixed effects logistic regression were performed for risk adjustment with covariates such as age, cancer occurrence, inflammatory bowel disease, American Society of Anesthesiologists physical status classification, perioperative VTE prophylaxis, postoperative VTE prophylaxis, surgical approach, and surgical duration, among other variables.

**Results:**

In total, 24,513 patients underwent hysterectomy. Of these patients, 1,107 (4.45%) received eVTEp, 153 (13.24%) of which were Black and 954 (82.53%) of which were White. Mixed effects logistic regression analysis suggested that Black patients were significantly less likely to receive eVTEp than White patients (odds ratio = 0.776; 95% CI: 0.615–0.979; P = 0.039). Additionally, tobacco use, coronary artery disease, bleeding disorder, cancer occurrence, functional status, perioperative VTE prophylaxis, surgical duration, length of stay, and surgical approach were associated with a higher likelihood of receiving eVTEp.

**Conclusion:**

eVTEp is recommended for the prevention of post-discharge VTE in select patients after hysterectomy. Regression analysis showed that, compared to their White counterparts, Black females were significantly less likely to receive eVTEp. The underlying reasons for this disparity require further investigation into possible socioeconomic influences and inherent biases.

## Introduction

Venous thromboembolism (VTE), comprised of deep vein thrombosis (DVT) and pulmonary embolism (PE), is a clotting disorder affecting the venous system of the body [1, 2]. VTE can have serious disabling health consequences and remains a leading cause of preventable hospital death. Previous studies suggest that 5%-10% of in-hospital deaths are due to PE [3, 4]. Additionally, recent data have shown that post-discharge VTE may account for as much as 75% of VTE occurrences, representing a large proportion of preventable postoperative morbidity and mortality [5–8]. Furthermore, with an estimated annual cost of $13.5-$27.2 billion in the United States for 375,000 to 425,000 newly diagnosed, medically treated incident cases, VTE poses a significant socioeconomic burden on the United States healthcare system [9–11]. Therefore, appropriate management of VTE is of paramount importance, contributing to patient safety and well-being, and cost-effective care [12].

Extended VTE prophylaxis (eVTEp) has been recommended for patients undergoing major abdominal or pelvic surgery, with a combination of both mechanical and pharmacologic prophylaxis showing the greatest reduction in VTE occurrence [13, 14]. Specifically, in women undergoing significant gynecologic surgery without prophylaxis, the risk of DVT varies from 17% to 40%. For those undergoing surgery specifically for gynecologic cancer, the risk of DVT is notably higher in the absence of thromboprophylaxis [15]. Currently, the administration of eVTEp depends upon each patient’s perceived VTE risk, which is based on preoperative and perioperative factors such as age, presence of cancer, and history of VTE. There are, however, no generally agreed-upon criteria for selecting patients for eVTEp [16–21].

While the prescription of prophylaxis ought to be determined by VTE risk factors rather than the social identities of patients, current literature suggests racial disparities may exist in post-operative care with regard to incidence of VTE [22–24]. Moreover, there is limited evidence regarding racial disparities in the prescription of post-discharge or extended prophylaxis. To determine if racial disparities exist in the administration of eVTEp after hysterectomy, we investigated the rate of eVTEp prescription rates with respect to race. The findings of this project have the potential to inform evidence-based health policies and produce targeted strategies aimed at reducing racial inequities in terms of access and receipt of risk-appropriate eVTEp.

## Methods

### Study design

A retrospective cohort study was conducted leveraging data abstracted from electronic medical records (EMRs) for 24,513 White and Black/African American patients who underwent hysterectomy between January 2016 and February 2020 in 69 academic and community hospitals (deidentified) across the State of Michigan. Data abstraction was done as part of the work of the Michigan Surgical Quality Collaborative (MSQC) and funded by the Blue Cross Blue Shield of Michigan Value Partnership Program. Eligible patients undergoing hysterectomy were identified using the following 23 Current Procedural Terminology Category I codes as of December 31, 2020: total abdominal hysterectomy (corpus and cervix), with or without removal of tube(s), with or without removal of ovary(s) (58150, 58152), hysterectomy procedures (58180, 58200, 58210), laparoscopy, surgical, supracervical hysterectomy, for uterus 250 g or less (58541, 58542, 58543, 58544), laparoscopic/hysteroscopic procedures on the corpus uteri (58548), laparoscopy, surgical, with vaginal hysterectomy, for uterus 250 g or less (58550, 58552), laparoscopy, surgical, with vaginal hysterectomy, for uterus greater than 250 g (58553, 58554), laparoscopy, surgical, with total hysterectomy, for uterus 250 g or less (58570, 58571), laparoscopy, surgical, with total hysterectomy, for uterus greater than 250 g (58572, 58573), laparoscopic/hysteroscopic procedures on the corpus uteri (58575), resection (initial) of ovarian, tubal or primary peritoneal malignancy with bilateral salpingo-oophorectomy and omentectomy (58951), bilateral salpingo-oophorectomy with omentectomy, total abdominal hysterectomy and radical dissection for debulking (58953, 58954), and excision procedures on the ovary (58956).

As determined by the University of Michigan Institutional Review Board, the MSQC data collection is considered ‘Not Regulated’ (HUM00073978) and does not meet the definition of human subject research.

### Variables

The primary outcome was the eVTEp administration. The variable of interest was race (White or Black/African American). Other variables for risk adjustment included age, body mass index (BMI), tobacco use, alcohol consumption, diabetes, hypertension, cancer occurrence, bleeding disorder, inflammatory bowel disease (IBD), coronary artery disease (CAD), congestive heart failure (CHF), preoperative sepsis, functional status, American Society of Anesthesiologists (ASA) physical status classification (independent, dependent, or unknown), perioperative VTE prophylaxis, postoperative VTE prophylaxis, surgical approach (minimally invasive versus open), personal history of VTE, family history of VTE, length of hospital stay (LOS), and duration of surgery (30–360 minutes or 361–480 minutes).

### Statistical analyses

We described the baseline sociodemographic and clinical characteristics and explored bivariate associations between the covariates and eVTEp administration, followed by another baseline comparison of unadjusted rates of VTE risk factors by race using Pearson’s chi-squared tests and student’s t-tests (Tables 1 and 2). VTE risk scores as defined by Pannucci et al. were calculated with the mean and standard deviation reported, respectively, for Black and White patients (Table 3) [20]. To account for the fact that patients were clustered by hospitals, we fit a mixed-effects logistic regression model with fixed effects of risk factors including race, and random hospital effects (Table 4). Adjusted odds ratios (aOR) and 95% confidence intervals (CI) were reported. Statistical analyses were conducted in R 3.6.3 (R Foundation for Statistical Computing, Vienna, Austria). Statistical significance was defined as a *p*-value less than 0.05.

**Table 1:** Baseline comparison of sociodemographic and clinical characteristics by extended venous thromboembolism (VTE) prophylaxis administration.

Characteristic	Extended VTE Prophylaxis (eVTEp)			Unadjusted OR (CI)	p-value
	Yes (n = 1107)	No (n = 23406)	Total (n = 24513)		
Age, n (%)					
18 to 34 y	55 (4.97%)	2597 (11.10%)	2652 (10.82%)	0.78 (0.58–1.02)	0.081
35 to 54 y	431 (38.93%)	15813 (67.56%)	16244 (66.27%)	Reference	-
55 to 74 y	501 (45.26%)	4488 (19.17%)	4989 (20.35%)	4.10 (3.59–4.68)	<0.001
75+ y	120 (10.84%)	508 (2.17%)	628 (2.56%)	8.67 (6.93–10.78)	<0.001
Race, n (%)					
Black	153 (13.82%)	3930 (16.79%)	4083 (16.66%)	0.79 (0.67–0.94)	0.010
White	954 (86.18%)	19476 (83.21%)	20430 (83.34%)	Reference	-
BMI, n (%)					
<18.5	7 (0.63%)	211 (0.90%)	218 (0.89%)	0.64 (0.27–1.26)	0.242
18.5–25	190 (17.16%)	4449 (19.01%)	4639 (18.92%)	0.82 (0.69–0.96)	0.018
25–30	247 (22.31%)	6026 (25.75%)	6273 (25.59%)	0.79 (0.68–0.91)	0.002
>30	663 (59.89%)	12720 (54.35%)	13383 (54.60%)	Reference	-
Diabetes, n (%)					
Yes	194 (17.52%)	2129 (9.10%)	2323 (9.48%)	2.12 (1.80–2.49)	<0.001
No	913 (82.48%)	21277 (90.90%)	22190 (90.52%)	Reference	-
CHF, n (%)					
Yes	4 (0.36%)	17 (0.07%)	21 (0.09%)	4.99 (1.43–13.51)	0.004
No	1103 (99.64%)	23389 (99.93%)	24492 (99.91%)	Reference	-
CAD, n (%)					
Yes	94 (8.49%)	342 (1.46%)	436 (1.78%)	6.26 (4.91–7.90)	<0.001
No	1013 (91.51%)	23064 (98.54%)	24077 (98.22%)	Reference	-
Bleeding disorder, n (%)					
Yes	67 (6.05%)	124 (0.53%)	191 (0.78%)	12.10 (8.89–16.33)	<0.001
No	1040 (93.95%)	23282 (99.47%)	24322 (99.22%)	Reference	-
Cancer, n (%)					
Yes	641 (57.90%)	2639 (11.27%)	3280 (13.38%)	10.82 (9.55–12.28)	<0.001
No	466 (42.10%)	20767 (88.73%)	21233 (86.62%)	Reference	-
IBD, n (%)					
Yes	0	0	0	–	
No	1107 (100%)	23406 (100%)	24513 (100.00%)		
ASA Physical Status, n (%)					
1 to 3	1084 (97.92%)	23280 (99.46%)	24364 (99.39%)	Reference	-
4 to 5	23 (2.08%)	126 (0.54%)	149 (0.61%)	3.92 (2.44–6.02)	<0.001
Functional status, n (%)					
Independent	1056 (95.39%)	23268 (99.41%)	24324 (99.23%)	Reference	-
Dependent	21 (1.90%)	106 (0.45%)	127 (0.52%)	4.37 (2.65–6.85)	<0.001
Unknown	30 (2.71%)	32 (0.14%)	62 (0.25%)	20.66 (12.46–34.16)	<0.001
Hypertension, n (%)					
Yes	509 (45.98%)	6622 (28.29%)	7131 (29.09%)	2.16 (1.91–2.44)	<0.001
No	598 (54.02%)	16784 (71.71%)	17382 (70.91%)	Reference	-
Smoking, n (%)					
Yes	163 (14.72%)	5694 (24.33%)	5857 (23.89%)	0.54 (0.45–0.63)	<0.001
No	944 (85.28%)	17712 (75.67%)	18656 (76.11%)	Reference	-
Alcohol consumption, n (%)					
Yes	10 (0.90%)	148 (0.63%)	158 (0.64%)	1.43 (0.70–2.59)	0.273
No	1097 (99.10%)	23258 (99.37%)	24355 (99.36%)	Reference	-
Perioperative VTE prophylaxis, n (%)					
Yes	1086 (98.10%)	22740 (97.15%)	23826 (97.20%)	Reference	
No	21 (1.90%)	666 (2.85%)	687 (2.80%)	0.66 (0.41–1.00)	0.064
Postoperative VTE prophylaxis, n (%)					
Yes	914 (82.57%)	18270 (78.06%)	19184 (78.26%)	Reference	
No	193 (17.43%)	5136 (21.94%)	5329 (21.74%)	0.75 (0.64–0.88)	<0.001
Preoperative sepsis, n (%)					
Yes	1107 (100%)	23400 (99.97%)	24507 (99.98%)	-	
No	0 (0%)	6 (0.03%)	6 (0.02%)		
Personal History of DVT/PE, n (%)					
Yes	231 (20.87%)	641 (2.74%)	872 (3.56%)	9.37 (7.93–11.03)	<0.001
No	876 (79.13%)	22765 (97.26%)	23641 (96.44%)	Reference	-
Family history of DVT/PE, n (%)					
Yes	29 (2.62%)	306 (1.31%)	335 (1.37%)	2.03 (1.35–2.93)	<0.001
No	1078 (97.38%)	23100 (98.69%)	24178 (98.63%)	Reference	-
Surgical time					
30 to 360 min	1080 (97.56%)	23298 (99.54%)	24378 (99.45%)	Reference	-
361 to 480 min	27 (2.44%)	108 (0.46%)	135 (0.55%)	5.39 (3.45–8.13)	<0.001
Length of stay, n (%)					
0 to 1 day	516 (46.61%)	17195 (73.46%)	17711 (72.25%)	Reference	-
2 to 4 days	417 (37.67%)	5731 (24.49%)	6148 (25.08%)	2.42 (2.12–2.77)	<0.001
5 to 9 days	147 (13.28%)	359 (1.53%)	506 (2.06%)	13.65 (11.03–16.82)	<0.001
10 to 30 days	27 (2.44%)	121 (0.52%)	148 (0.60%)	7.44 (4.76–11.21)	<0.001
Surgical approach, n (%)					
Minimally invasive	603 (54.47%)	18126 (77.44%)	18729 (76.40%)	0.35 (0.31–0.39)	<0.001
Open	504 (45.53%)	5280 (22.56%)	5784 (23.60%)	Reference	-
Insurance, n (%)					
HMO	246 (22.22%)	6596 (28.18%)	6842 (27.91%)	0.73 (0.63–0.84)	<0.001
Commercial non-HMO	412 (37.22%)	11541 (49.31%)	11953 (48.76%)	Reference	-
Government/Medicaid	416 (37.58%)	5053 (21.59%)	5469 (22.31%)	2.19 (1.93–2.48)	<0.001
Other/uninsured/self-pay	33 (2.98%)	216 (0.92%)	249 (1.02%)	3.30 (2.24–4.71)	<0.001

Notes: BMI, Body Mass Index; CHF, congestive heart failure; CAD, coronary artery disease; IBD, inflammatory bowel disease; DVT, deep vein thrombosis; PE, pulmonary embolism; HMO, health maintenance organization; OR, odds ratio; alcohol consumption indicates greater than 2 drinks per day in the 2 weeks prior to admission.

**Table 2:** Baseline comparison of venous thromboembolism (VTE) risk factors by race.

Characteristic	Race			Unadjusted odds ratio (CI) / coefficient (CI)	p-value
	White (n = 20430)	Black (n = 4083)	Total (n = 24513)		
Age, n (%)					
> = 60	3548 (17.37%)	392 (9.60%)	3940 (16.07%)	0.51 (0.45–0.56)	<0.001
<60	16882 (82.63%)	3691 (90.40%)	20573 (83.93%)	Reference	-
BMI, n (%)					
> = 40	3005 (14.71%)	868 (21.26%)	3873 (15.80%)	1.57 (1.44–1.70)	<0.001
<40	17425 (85.29%)	3215 (78.74%)	20640 (84.20%)	Reference	-
Preoperative sepsis, n (%)					
Yes	20425 (99.98%)	4082 (99.98%)	24507 (99.98%)	1.00 (0.16–19.15)	0.999
No	5 (0.02%)	1 (0.02%)	6 (0.02%)	Reference	-
Personal history of DVT / PE, n (%)					
Yes	705 (3.45%)	167 (4.09%)	872 (3.56%)	1.19 (1.00–1.41)	0.044
No	19725 (96.55%)	3916 (95.91%)	23641 (96.44%)	Reference	-
Family history of DVT / PE, n (%)					
Yes	283 (1.39%)	52 (1.27%)	335 (1.37%)	0.92 (0.67–1.23)	0.575
No	20147 (98.61%)	4031 (98.73%)	24178 (98.63%)	Reference	-
Cancer, n (%)					
Yes	2952 (14.45%)	328 (8.03%)	3280 (13.38%)	0.52 (0.46–0.58)	<0.001
No	17478 (85.55%)	3755 (91.97%)	21233 (86.62%)	Reference	-
Smoking, n (%)					
Yes	4877 (23.87%)	980 (24.00%)	5857 (23.89%)	1.01 (0.93–1.09)	0.859
No	15553 (76.13%)	3103 (76.00%)	18656 (76.11%)	Reference	-
Diabetes, n (%)					
Yes	1830 (8.96%)	493 (12.07%)	2323 (9.48%)	1.40 (1.25–1.55)	<0.001
No	18600 (91.04%)	3590 (87.93%)	22190 (90.52%)	Reference	-
CHF, n (%)					
Yes	16 (0.08%)	5 (0.12%)	21 (0.09%)	1.56 (0.51–4.00)	0.383
No	20414 (99.92%)	4078 (99.88%)	24492 (99.91%)	Reference	-
Hypertension, n (%)					
Yes	5321 (26.05%)	1810 (44.33%)	7131 (29.09%)	2.26 (2.11–2.42)	<0.001
No	15109 (73.95%)	2273 (55.67%)	17382 (70.91%)	Reference	-
Surgical time (hour)					
Mean (SD)	2.03 (0.98)	2.51 (1.02)	2.11 (1.00)	0.48 (0.45–0.52)	<0.001
Median [Min, Max]	1.81 [0.50, 8.00]	2.32 [0.65, 7.88]	1.90 [0.50, 8.00]	-	-
Length of stay (day)					
Mean (SD)	1.22 (1.93)	1.92 (1.85)	1.34 (1.94)	0.70 (0.63–0.76)	<0.001
Median [Min, Max]	1.00 [0, 30.0]	2.00 [0, 30.0]	1.00 [0, 30.0]	-	-

Notes: BMI, Body Mass Index; CHF, congestive heart failure; CAD, coronary artery disease; DVT, deep vein thrombosis; PE, pulmonary embolism; CHF, congestive heart failure. VTE risk factors were recorded according to Pannucci et al. [20].

**Table 3:** Summary of Pannucci score by race.

Pannucci score (n = 24513)	Race			*P*-value
	White (n = 20430)	Black (n = 4083)	Total (n = 24513)	<0.001
Mean (SD)	4.20 (2.17)	3.88 (1.79)	4.15 (2.11)	
Median [Min, Max]	3 [0, 16]	3 [1, 14]	3 [0, 16]	

**Table 4:** Multivariable mixed effect logistic regression model of extended venous thromboembolism (VTE) prophylaxis administration.

Characteristic (n = 24513)	Adjusted OR/Coefficient	95% CI	p-value
Age			
18 to 34 y	1.021	(0.742–1.404)	0.900
35 to 54 y	Reference	-	-
55 to 74 y	1.305	(1.081–1.575)	0.005
75+ y	1.377	(0.992–1.911)	0.056
Race			
Black	0.776	(0.615–0.979)	0.032
White	Reference	-	-
BMI			
<18.5	0.627	(0.248–1.584)	0.323
18.5–25	0.890	(0.721–1.097)	0.275
25–30	0.942	(0.787–1.129)	0.519
>30	Reference	-	-
Diabetes			
Yes	0.980	(0.793–1.213)	0.855
No	Reference	-	-
CHF			
Yes	1.558	(0.419–5.803)	0.508
No	Reference	-	-
CAD			
Yes	4.481	(3.239–6.200)	<0.001
No	Reference	-	-
Bleeding disorder			
Yes	12.806	(8.426–19.462)	<0.001
No	Reference	-	-
Cancer			
Yes	5.687	(4.700–6.881)	<0.001
No	Reference	-	-
ASA Physical Status			
1 to 3	Reference	-	-
4 to 5	0.852	(0.468–1.549)	0.598
Functional status			
Independent	Reference	-	-
Dependent	1.793	(0.962–3.340)	0.066
Unknown	7.775	(4.279–14.128)	<0.001
Hypertension			
Yes	1.068	(0.902–1.265)	0.442
No	Reference	-	-
Smoking			
Yes	0.795	(0.649–0.975)	0.027
No	Reference	-	-
Alcohol consumption			
Yes	1.683	(0.784–3.611)	0.181
No	Reference	-	-
Perioperative VTE prophylaxis			
Yes	Reference	-	-
No	0.577	(0.349–0.954)	0.032
Postoperative VTE prophylaxis			
Yes	Reference	-	-
No	1.007	(0.798–1.270)	0.955
Personal History of DVT/PE			
Yes	9.832	(7.892–12.250)	<0.001
No	Reference	-	-
Family history of DVT/PE			
Yes	1.027	(0.634–1.664)	0.914
No	Reference	-	-
Surgical time (hours)	1.175	(1.093–1.264)	<0.001
Length of stay			
0 to 1 day	Reference	-	-
2 to 4 days	1.582	(1.218–2.056)	0.001
5 to 9 days	2.287	(1.571–3.330)	<0.001
10 to 30 days	1.383	(0.745–2.567)	0.305
Surgical approach			
Minimally invasive	0.512	(0.396–0.662)	<0.001
Open	Reference	-	-
Insurance			
HMO	1.142	(0.943–1.384)	0.174
Commercial non-HMO	Reference	-	-
Government/Medicaid	1.185	(0.986–1.423)	0.070
Other/uninsured/self-pay	1.864	(1.167–2.976)	0.009

Notes: BMI, Body Mass Index; CHF, congestive heart failure; CAD, coronary artery disease; DVT, Deep Vein Thrombosis; PE, Pulmonary Embolism; HMO, health maintenance organization; ASA, American Society of Anesthesiologists; alcohol consumption indicates greater than 2 drinks per day in the 2 weeks prior to admission.

## Results

From January 2016 to February 2020, the MSQC collected data on 24,513 White and Black patients who underwent hysterectomy. Patients were divided based on the presence (1,107) or absence (23,406) of eVTEp administration. Of the group that received eVTEp, 954 (86.18%) were White and 153 (13.82%) were Black.

Baseline characteristics of all patients by eVTEp prescription are displayed in Table 1. There was a higher frequency of eVTEp prescription in patients older than 55 years of age, BMI > = 30, history of diabetes, CHF, CAD, bleeding disorder, cancer, ASA status >3, dependent or unknown functional status, history of hypertension, administration of postoperative VTE prophylaxis, personal history of VTE, family history of VTE, surgical time >360 minutes, LOS >1 day, minimally invasive surgical approach, and government/Medicaid or other health insurance.

Table 2 compares the frequency of VTE risk factors by race. Black patients are noted to have a greater frequency of BMI > = 40, history of diabetes, hypertension, longer surgical time, and length of stay, as compared to their White counterparts. Comparatively in this cohort, White patients were noted to have a greater frequency of age > = 60 and personal history of cancer.

Based on the cohort characteristics in Table 2, a score was calculated based on Pannucci et al. and presented in Table 3. White patients had an average risk score of 4.20 (SD = 2.17) as compared to Black patients with an average risk score of 3.88 (SD = 1.79), with a p-value of <0.001 indicating a statistically significant difference between scores.

The results of multivariable logistic regression analyses are summarized in Table 4. In the mixed-effects model adjusting for risk factors across hospitals, Black patients had approximately 25% lower odds (aOR = 0.776, 95% CI = 0.615–0.979) of receiving eVTEp than the White patients. Significant associations were also revealed between lower occurrence of eVTEp prescription and smoking history (aOR = 0.795, 95% CI = 0.649–0.975), perioperative VTE prophylaxis (aOR = 0.577, 95% CI = 0.349–0.954), and invasive surgical approach (aOR = 0.512, 95% CI = 0.396–0.662). In contrast, higher odds of eVTEp prescription were significantly associated with age 55–74 (aOR = 1.305, 95% CI = 1.081–1.575), CAD (aOR = 4.481, 95% CI = 3.239–6.200), bleeding disorder (aOR = 12.806, 95% CI = 8.426–19.462), unknown functional status (aOR = 7.775, 95% CI = 4.279–14.128), cancer (aOR = 5.687, 95% CI = 4.700–6.881), personal history of DVT/ PE (aOR = 9.832, 95% CI = 7.892–12.250), longer surgical time (coefficient = 1.175, 95% CI = 1.093–1.264), LOS of 2–9 days (aOR = 1.582, 95% CI = 1.218–2.056 for 2–4 days and aOR = 2.287, 95% CI = 1.571–3.330 for 5–9 days) and other insurance/uninsured/self-pay (aOR = 1.864, 95% CI = 1.167–2.976).

## Discussion

For most patients undergoing benign gynecologic surgery, mechanical prophylaxis is appropriate for prevention of VTE [25]. However, pharmacological VTE prophylaxis may be beneficial for many patients such as those requiring prolonged immobilization or for gynecologic oncology patients the risk of VTE may persist well beyond 4 weeks [26, 27]. In this study, we sought to identify the relationship between race and eVTEp prescription after hysterectomy surgery. We found that Black patients were prescribed eVTEp at lower rates as compared to their White counterparts, even after risk adjustment with multivariable logistic regression.

There are no broadly accepted guidelines for prescribing eVTEp, and the etiology of this discrepancy is likely multifactorial. Prior studies suggest Black Americans are at a higher risk for VTE as compared to their White counterparts due to a higher prevalence of diabetes, obesity, elevated factor VIII, and genetic polymorphisms in genes coding for prothrombin [23, 28–30]. Indeed, this study’s findings correspond with prior literature as the Black cohort was noted to have a significantly greater proportion of obesity, diabetes, and prior VTE history as compared to their White counterparts. However, among the White cohort, this study noted significantly increased proportions of patients over the age of 75 and with cancer, variables classically associated with increased VTE incidence. Within this study, the incidence of post-discharge VTE was low (0.26% for PE, 0.12% for DVT). While not statistically significant, the proportion of patients with post-operative VTE was higher in the Black cohort (0.37% for PE, 0.17% for DVT) as compared to the White cohort (0.23% for PE, 0.11% for DVT).

Variations in the prescription of eVTEp may be attributed to many factors including, but not limited to, risk scores, physician clinical judgement, regional practices, and hospital protocol. In regard to VTE risk, this study identified a significant difference in the Pannucci VTE risk score between White and Black patients, with the former cohort having a higher average risk score (4.20 vs 3.88). Though the White cohort had a statistically significantly higher average risk score, this does not translate to clinical significance. The Pannucci VTE risk score is on a whole-point scale, and with the cohorts’ averages rounded to the nearest whole number, there would be no difference in risk score–both the Black and White cohorts would have a risk score of 4. Further, the Pannucci VTE risk score is on a 17-point scale, even if there were a whole-point difference in score, the risk score would categorize them in the same risk category (not high-risk). Taken together, the lack of significant clinical difference in risk scores between the White and Black cohorts and the higher prevalence of certain risk factors in the Black cohort necessitates a change in eVTEp prescription practices.

The analysis boasts a large cohort of 24,513 female patients undergoing hysterectomy with comprehensive risk adjustment. Consequently, significant disparities were identified regarding the administration of eVTEp with sufficient statistical power. Due to its retrospective design, the findings are susceptible to potential coding errors, missing data, and data entry mistakes. Additionally, there was a lack of information including specific indications for eVTEp, the specific eVTEp regimens prescribed, and patient compliance with prescriptions. While there was information regarding post-operative VTE, post-discharge VTE events and outcomes were not available. Lastly, provider-related causes such as surgeon sex, surgeon education, surgeon experience, and surgeon race, potentially having an impact on the disparities of the administration of eVTEp, were not available in the data.

## Conclusions

This large retrospective cohort study showed a lower rate of prescription of eVTEp in Black patients compared to White patients after certain gynecological operations despite no clinically significant difference in risk scores between the cohorts. This study identifies a unique difference in care regarding risk-appropriate prophylaxis treatment among patients undergoing gynecologic surgery by racial group. While risk scores may be employed by physicians, this study suggests a need to explore eVTEp prescription practices in greater detail to ensure safe and equitable care is provided to all patients regardless of race.
